# Designing an Efficient and Quality-focused Integrated Atrial Fibrillation Care Center

**DOI:** 10.19102/icrm.2022.13107

**Published:** 2022-10-15

**Authors:** Joshua Silverstein, Paul D. Varosy, Brigham Godfrey, Christopher Cooper, Allyson Varley, Anil Rajendra, Gustavo Morales, Jose Osorio

**Affiliations:** ^1^Mount Carmel Health System, Columbus, OH, USA; ^2^Allegheny Health Network, Pittsburgh, PA, USA; ^3^VA Eastern Colorado Health Care System and University of Colorado Anschutz Medical Campus, Aurora, CO, USA; ^4^Arrhythmia Institute at Grandview, Birmingham, AL, USA; ^5^Heart Rhythm Clinical and Research Solution, Innovation Depot, Birmingham, AL, USA

**Keywords:** Atrial fibrillation, atrial fibrillation center of excellence, catheter ablation, quality improvement

## Abstract

Atrial fibrillation (AF) represents a significant health care burden in the United States that will continue to increase as the population ages; thus, the introduction of cost-effective strategies to limit this burden is critical. The establishment of dedicated electrophysiology programs focusing on AF care within hospitals can improve patient care while providing added financial benefits for institutions if properly planned and delivered. This paper explains how to develop an efficient and quality-focused AF ablation program as part of a larger AF center of excellence by highlighting the experience of a single center and demonstrating how the same principles were adopted to implement a similar program at another institution.

## Introduction

The prevalence of atrial fibrillation (AF) continues to rise, and, in turn, the demand for dedicated electrophysiology (EP) programs is increasing. One in four adults aged >40 years are at risk of developing AF, and 5.5 million Americans currently live with AF.^1^ An AF diagnosis can lead to other heart-related complications, including stroke and heart failure.^2^ AF represents a significant health care burden in the United States that will continue to increase as the population ages.^3,4^ Thus, cost-effective strategies to limit this burden are critical.

While most hospital cardiology service lines are well established, many lack an integrated EP program with a focus on AF-specific care (ie, an AF center of excellence [CoE]) to streamline and improve outcomes. The establishment of dedicated EP programs with a focus on AF care within hospitals can improve patient care while providing added financial benefits for institutions if properly planned and delivered. The purpose of this paper was to delineate how to develop an efficient and quality-focused AF ablation program as part of a larger AF CoE. The paper will highlight the experience of a single center and demonstrate how the same principles were adopted to implement a similar program at another institution.

## The case for integrated atrial fibrillation centers of excellence

The treatment of AF is rapidly changing. Over the past decade, there has been an increasing emphasis on lifestyle and risk factor modification, stroke prophylaxis, and the use of catheter ablation as a first-line therapy for some patients. Moreover, the expeditious identification of patients and the initiation of appropriate therapy in the emergency department can reduce hospital stay and improve outcomes.^5^ Therefore, there has been a growing need for specialized, integrated EP programs that focus on AF care, quality improvement (QI), and efficiency. AF clinics and protocols that integrate emergency department, hospital, and outpatient care can not only streamline patient care but also ensure access to the appropriate guideline-recommended practices, such as stroke prophylaxis, rhythm control, and lifestyle modification.^6^ The Heart Rhythm Society published a perspective piece in 2020 regarding the importance of developing an AF CoE to improve the quality of care and access for patients with AF.^7^

## Benefits of atrial fibrillation ablation

Randomized trials demonstrate a reduction in arrhythmia burden with AF ablation compared to anti-arrhythmic drug (AAD) therapy for both paroxysmal and persistent AF.^8^ Recent data from the Atrial Fibrillation Progression Trial (ATTEST) report that catheter ablation may be nearly 10 times more effective than drug therapy alone at delaying AF progression.^9^ Catheter ablation has also been shown to be superior to the AAD amiodarone in achieving freedom from AF at long-term follow-up and in reducing unplanned hospitalizations and mortality in patients with heart failure and persistent AF.^10^ The Catheter Ablation for Atrial Fibrillation with Heart Failure (CASTLE AF) study found a reduction in the composite endpoint of death from any cause or hospitalization for heart failure in patients with pre-existing congestive heart failure.^11^ Patients with pre-existing cardiac conditions who were treated earlier with a rhythm-control strategy compared to a rate-control strategy in the Early Treatment of Atrial Fibrillation for Stroke Prevention Trial (EAST-AFNET4) had a lower risk of adverse cardiovascular outcomes.^12^ Catheter ablation for AF can allow patients to live symptom-free without postoperative AAD therapy.^13^ Moreover, the initiation of some AAD therapies requires longer inpatient stays than catheter ablation does. Based on this growing body of evidence, the recommendations for AF treatment have changed, and industry forecasts predict that catheter ablation procedures will increase by 10%–12% per year over the next 5 years.^14^

There is an unmet need for AF treatment and EP expertise within the community as well as hospitals’ service lines to avoid preventing patients from receiving guideline-recommended therapies that could lead to better outcomes.^15^ Recent guidelines recommend AF ablation as a first-line therapy for some patients, thereby significantly increasing the number of patients who are eligible for the treatment.^16–19^ Catheter ablation, with or without AAD therapy for symptomatic or drug-refractory paroxysmal AF, is more cost-effective than AAD therapy alone in terms of both improvements in quality of life and avoidance of future health care costs.^20^ However, many institutions fall short when implementing new guidelines because they lack dedicated EP programs, have programs operating at capacity, or do not have an integrated AF care solution. These factors limit an institution’s ability to handle the potential increase in AF treatment demand.

## The atrial fibrillation patient journey: can we improve it with integrated atrial fibrillation care centers?

Institutions lacking an integrated AF care center are likely to experience issues with continuity, leading to unnecessary delays in the time from diagnosis to treatment. One study indicated that the median time from emergency department visit to EP assessment was 87 days and the median time from EP assessment to ablation procedure was approximately 300 days.^21^ It can take nearly a year for a patient to receive an AF ablation after their initial emergency department visit. As the time to see a specialist and receive appropriate treatment increases, outcomes may be impacted. AF ablation has the highest success rate when the procedure is performed <24 months after initial symptoms.^22^

Therefore, delays in specialized evaluation and care can impact not only patient outcomes but also hospital and overall health care costs. Patients with recurrent symptomatic AF may be more likely to have additional emergency department visits, hospital admissions, and health care utilization while waiting to be seen by an electrophysiologist.^23^ When AF is efficiently treated with specialized evaluation and proper use of stroke prophylaxis and rhythm-control strategies, higher success rates are achieved, leading to better outcomes and decreased costs.^12,24^

## Developing an efficient electrophysiology program and atrial fibrillation center of excellence

There is increasing interest in a model that would create specialized, efficient, and high-quality centers that deliver care around a single disease state and result in more patient-centered care.^25–28^ However, such programs require a dedicated team of specialists, QI processes, inter-department integration, and administrative support. Careful financial considerations for the creation of sustainable and efficient programs are also needed.

This is an important consideration as hospitals make strategic decisions on how to design and create a center to better care for patients with AF. The benefits of an AF care program that integrates the hospital and outpatient settings are shown in **Table 1**.

As one considers the increase in the number of patients with AF and the design of a new AF CoE that integrates care across an institution, it is paramount to focus on quality, patient outcomes, and protocols to standardize care. This can only be achieved through a rigorous QI process.

### Focus on quality improvement—the foundation of developing an atrial fibrillation center of excellence

QI is a systematic, formal approach to the analysis of practice performance and efforts to improve it. Continuous QI means opportunities for improvement exist in every process.^29^ There are several approaches to QI frequently used in health care to understand variation, design, and implementation, such as Plan–Do–Act, Six Sigma, Lean, and Lean Six Sigma.^30^ Some approaches can be labor-intensive and lead to paralysis by analysis. In our opinion, however, there are simpler ways to increase the QI capacity and promote a culture of continuous improvement.^31,32^

When Grandview Medical Center opened in Birmingham, AL, in October 2015, there was a unique chance to create an EP and AF program from the ground up. The following strategic decisions were made to create the ethos of ever-improving quality:

1. Hospital administration, physician leadership, and EP laboratory staff alignment
2. Agreement among all parties that there would be financial, personnel, and time investments to create a high-quality program
3. Creation of protocols and standards for every aspect of patient flow and care, from arrival to patient preparation, and handling of complications or other emergencies
4. Establishment of benchmarks and committing to measuring patient flow in the hospital as well as outcomes
5. Collection of data from each procedure as part of an internal AF outcome registry to facilitate measurement of and demonstrate quality outcomes
6. Creation of an EP quality committee with representation from all stakeholders, including EP doctors, nurses, technicians, managers, service line directors, and administration
7. Choosing an accreditation program that allows for the comparison and benchmarking of the program against others nationally with a goal of continuous improvement

### Gather data—perform a deep dive into the patient experience

The next step was to understand the process involved in the care of each patient presenting to the hospital for an elective ablation procedure. A detailed analysis of the EP program was completed by administration and physician leadership to identify issues preventing the program from reaching its full potential. Only with a complete understanding of every step included in the patient journey (from arrival through discharge) could processes be designed and improved.

### Go over assessment and come up with solutions as a team

The EP laboratories were understaffed, and there were inefficiencies in the laboratory that led to longer procedures and late days. The combination of a staffing shortage, inefficiencies, and long days led to staff burnout and higher turnover. Once a detailed assessment of the EP program was completed, the administration, physicians, and laboratory staff engaged in developing plans to overcome these challenges. The administration committed to hiring an adequate number of staff dedicated to specializing in EP. Protocols were developed to improve the quality and safety of patients and staff.

A primary goal was to improve efficiency by standardizing every step of the patient journey from the day of the procedure. Starting from registration to patient discharge, the entire patient journey was mapped. Protocols and time expectations were agreed upon with the participation of all stakeholders.

### Measure performance

The process was continuously monitored to ensure that each team was meeting the agreed-upon standards, which then became the protocol. To maintain the efficiency achieved, several time points were continuously monitored, allowing the identification of areas for improvement **(Table 2)**.

Monitoring these time points could be accomplished using different information management systems available in EP and catheterization laboratories, which can be customized to each hospital’s specific needs and workflows. Monthly reports can be generated, which can be used for QI, to help understand staffing needs, and to help maintain a high level of efficiency.

### Form a quality improvement committee, meet regularly, learn from complications, and set goals

The QI committee was tasked with overhauling the identification and monitoring of complications. Event reporting systems are now used, and nurses throughout the hospital have been educated on the need to report procedure-related complications not just for the index admission but also for readmissions. All complications are then reviewed during meetings, and mitigation strategies are discussed with all clinicians. Complications are benchmarked according to historical rates for the patient group and compared to a large multisite registry.^33^

Another goal was reductions in the use of fluoroscopy and radiation exposure among patients and staff. The risk of radiation is often underappreciated in EP laboratories. The burden of wearing lead to protect staff members from radiation is known to cause orthopedic injuries. Protocols were developed and adopted by all electrophysiologists to minimize the use of fluoroscopy to such a level that it is no longer necessary for staff to wear lead during most procedures, leading to greater working environment satisfaction among EP staff.^34^ Protocols for reducing, monitoring, and reporting radiation exposure during implantation of devices for left atrial appendage (LAA) closure and cardiac rhythm management were reviewed by the QI committee.

Additionally, protocols were also developed for anesthesia during AF ablation, management of complications in the EP laboratory, and management of patients throughout the course of their treatment. All physicians who operate at Grandview Medical Center are expected to utilize the same protocols for their patients and during AF ablations to prevent complications and improve the quality of care.^35,36^

As AF ablation procedures became shorter and safer, we created protocols focusing on same-day discharge.^37^ We chose to start with a subset of patients identified as being at low risk for procedural and anesthetic complications (ie, on stable anticoagulation with no bleeding history, systolic heart failure, respiratory conditions, or interventional procedures within 60 days and a recommended body mass index of <35 kg/m^2^). Patients were identified in the clinic as they were being scheduled for ablation, with their procedures planned to be done earlier in the day. We demonstrated the safety of our protocol and have subsequently implemented same-day discharge for all patients undergoing AF ablation and LAA occlusion who safely complete the procedure and are clinically stable 5 h after the procedure. We have also found that proper anesthetic management^35,36^ and a focus on reducing the time to extubation have improved patient satisfaction and the likelihood of being discharged home the same day.

### Obtain electrophysiology laboratory accreditation

In 2016, Grandview Medical Center’s EP program in Columbus, OH, achieved Intersocietal Accreditation Commission (IAC) accreditation for their EP laboratory. IAC accreditation was a vehicle by which appropriate protocols were updated and created and QI programs were developed.^38^ The EP quality committee ensured that changes were maintained. This committee ensures that protocols are current, monitors QI projects, and reviews outcomes and complications.

As a result of these activities, Grandview’s EP program has grown to be a high-volume EP center with a 19% increase in laboratory volume from 2017 to 2018.^39^ In 2018, Grandview performed >2,500 procedures, which reached >3,000 in 2021—including 1,500 ablations (1,200 of which were AF ablations), 900 implantations of cardiac rhythm management devices, 300 LAA closures, and 130 lead extractions. More than 400 electrophysiologists have visited the hospital to observe the efficient laboratory operation and learn about fluoroscopy reduction and new technologies and techniques in AF ablation.

## Is the process reproducible?

Dr. Joshua Silverstein, of Mount Carmel Medical Group at the time, visited Grandview twice during 2015–2016. The goal of both visits was to improve EP laboratory efficiency. Two EP laboratory nurses also attended the first visit in 2015. During the first visit, Dr. Silverstein and his team were overwhelmed by what they saw and did not think that they could reproduce the model. However, they took extensive notes of what they observed. After the trip to Grandview, Dr. Silverstein, both nurses, and an administrator met with their team to discuss their observations and to determine what steps could be implemented in their laboratory.

Before the initial visit of Dr. Silverstein’s team to Grandview, Mount Carmel’s EP program performed a maximum of 2 AF catheter ablations per day. The procedural and operational changes made upon returning to Mount Carmel allowed their program to increase to performing 3 ablations per day within the first several months. To achieve this, the Mount Carmel team focused on systematically improving procedural and periprocedural efficiency.

The team’s second visit to Grandview included a hospital administrator, 2 additional nurses, and another EP physician and was used to demonstrate the benefits of an additional EP laboratory. With an additional laboratory, Mount Carmel’s EP program was able to double the number of procedures completed a day, reduce procedure times from 3 to 1.5 h, and reduce wait times by half. The implemented QI strategies also provided an opportunity for more staff training and better team workflow. Mount Carmel’s EP program now hosts physicians interested in improving quality, safety, and efficiency in their EP laboratories. The changes implemented at Mount Carmel resulted in benefits to both patients and the hospital. In May 2021, Dr. Silverstein and one of the nurses who attended Grandview in 2014 had the opportunity to visit again. This time, instead of being overwhelmed by what they observed, they noticed how similar the 2 programs have become. Similar to Grandview, Mount Carmel’s EP laboratory also became accredited by the IAC.

This quality-focused collaboration has since evolved into a larger grassroots community of electrophysiologists participating in the Real-AF registry. In 2018, Grandview became the first active site in the registry (with Jose Osorio as the principal investigator).^33^ Soon after, in 2019, Mount Carmel became the second site to join the registry. Since 2019, the registry has grown to include 30 additional quality-driven sites with >58 electrophysiologists sharing data and best practices.

## Conclusion

The demand for EP services will continue to increase with the expected rise in AF incidence. Thus, more dedicated AF CoEs are necessary. The value of an efficient EP program within institutions outweighs the effort and cost of initial setup. An efficient EP program likely results in better patient outcomes as well as greater staff satisfaction and retention. Working in collaboration, EP programs can utilize accreditation programs, registries, and standardized workflows and procedures to help improve the quality and efficiency of EP laboratories.

## Figures and Tables

**Table 1:** Benefits of an Integrated Atrial Fibrillation Center of Excellence

1.	Specialized, high-volume institutions have been shown to deliver better care, with significantly lower complication rates^40,41^
2.	Integrating AF care from the ED to the hospital and outpatient clinics will reduce the time from diagnosis to treatment and facilitate the patient journey
3.	AF clinics improve access to outpatient care, reducing the need for hospital/ED visits
4.	Focused data collection and quality improvement
5.	Patient-centered with easier access and a focus on lifestyle modification and other therapies that impact outcomes of AF patients (like screening and referral to sleep centers)
6.	Easy access to comprehensive, multidisciplinary AF patient care offering the whole spectrum of treatment options (eg, surgical ablation specialist, hybrid ablation techniques, structural heart program for mitral valve disease, left atrial appendage occlusion)

*Abbreviations:* AF, arial fibrillation, ED, emergency department.

**Table 2:** EP Laboratory Time Points Monitored to Measure Efficiency

1.	Patient arrival to registration
2.	Patient arrival to pre-recovery area
3.	Patient ready for interaction with physician, electrophysiologist for consent, and anesthesia for assessment
4.	Anesthesiologist evaluation completed
5.	Patient arrival to the EP laboratory
6.	Patient prepped and ready for procedure
7.	Electrophysiologist arrival to the EP laboratory and procedure start time
8.	Procedure end time
9.	Patient extubation and anesthesia recovery
10.	Patient departs from the EP laboratory to recovery area

*Abbreviation:* EP, electrophysiology.
